# Effects of lifestyle risk behaviour clustering on cardiovascular disease among UK adults: latent class analysis with distal outcomes

**DOI:** 10.1038/s41598-022-22469-6

**Published:** 2022-10-17

**Authors:** Teketo Kassaw Tegegne, Sheikh Mohammed Shariful Islam, Ralph Maddison

**Affiliations:** 1 Institute for Physical Activity and Nutrition, Deakin University, Geelong, VIC Australia; 2 Department of Public Health, College of Health Sciences, Debre Markos University, Debre Markos, Ethiopia

**Keywords:** Cardiology, Diseases, Health care, Medical research, Risk factors, Statistical methods

## Abstract

Lifestyle risk behaviours such as smoking, physical inactivity, and unhealthy diet account for a considerable disease burden globally. These risk behaviours tend to cluster within an individual, which could have detrimental health effects. In this study, we aimed to examine the clustering effect of lifestyle risk behaviours on cardiovascular disease (CVD) and CVD risk among adults in the United Kingdom (UK). We performed a latent class (LC) analysis with distal outcomes using the UK Biobank baseline (2006–2010) data. First, we estimated LC measurement models, followed by an auxiliary model conditional on LC variables. We reported continuous (mean difference—MD) and binary (odds ratio—OR) outcomes with 95% confidence intervals. We included 283,172 and 174,030 UK adults who had data on CVD and CVD risk, respectively. Multiple lifestyle risk behaviour clustering (physically inactive, poor fruit & vegetable intake, high alcohol intake, and prolonged sitting) had a 3.29 mean increase in CVD risk compared to high alcohol intake. In addition, adults with three risk behaviours (physically inactive, poor fruit & vegetable intake, and high alcohol intake) had 25.18 higher odds of having CVD than those with two risk behaviours (physically inactive, and poor fruit and vegetable intake). Social deprivation, gender and age were also associated with CVD. Individuals' LC membership with two or more lifestyle risk behaviours negatively affects CVD. Interventions targeting multiple lifestyle behaviours and social circumstances should be prioritized to reduce the CVD burden.

## Introduction

Noncommunicable diseases (NCDs) are responsible for 71% of all deaths worldwide^1^. Lifestyle risk behaviours such as smoking, physical inactivity, alcohol consumption, and unhealthy diet increase the risk of dying from NCD^1^. Cardiovascular diseases (CVDs)^1–3^ account for most NCD deaths globally. In the UK, 11% of the population live with CVD, and CVD accounts for 25% of all deaths^4^. In 2010, the American Heart Association (AHA) recommended seven cardiovascular health behaviours (Life’s Simple 7) to reduce CVD morbidity and mortality in the general population, including smoking, diet, physical activity, body mass index, blood pressure, total cholesterol, and fasting glucose^5^. The 2019 AHA guideline on the primary prevention of CVD recommends that people engage in a healthy lifestyle throughout their life^6^.

Lifestyle risk behaviours, such as smoking, alcohol consumption, poor diet and physical inactivity are major risk factors for the development of CVD^7,8^. Physical inactivity and increased sedentary time are important modifiable risk factors for cardiometabolic disease^9,10^. Physical inactivity is also the fourth leading cause of disease and disability in the UK^11^. Sleeping too much or too little is also strongly associated with CVD^12–14^. People engaging in multiple risk behaviours tend to have poor health outcomes compared to those engaging in one risk behaviour^15^. Thus, identifying people with multiple risk behaviours provides insight into where policies should target to reduce inequalities in health^16,17^. While previous studies have investigated the clustering of lifestyle behaviours for people with CVD^18,19^ and the general population^20^, most did not include all major lifestyle behaviours, such as smoking, alcohol intake, fruit and vegetable consumption, physical activity, sitting behaviour, and sleep.

To analyse the co-occurrence of multiple health-related behaviours, different statistical approaches have been proposed in the literature^21^. However, these approaches are mainly focused on identifying clustering of risk behaviours and not estimating their effect on a distal outcome. Latent class (LC) analysis with a distal outcome is important for identifying how different lifestyle risk behaviours occur together among participants based on indicator variables, and to estimate the effect of LC membership on a distal outcome^22–24^. The effects of LC membership (clustering of lifestyle risk behaviours) on CVD and the risk of developing CVD have not yet been investigated. This study aimed to examine the prevalence of six lifestyle risk behaviours (smoking, poor fruit and vegetable consumption, alcohol intake, physical inactivity, prolonged sitting, and poor sleep), and clustering patterns of these lifestyle risk behaviours. In addition, we aimed to identify and estimate the effect of socio-demographic characteristics (age, gender), Townsend deprivation index and LC membership on CVD, and CVD risk.

## Methods

UK Biobank has ethics approval from the North West Multi-centre Research Ethics Committee^25^. According to this approval, researchers do not require separate ethics applications. All participants provided written informed consent. This study was carried out in accordance with relevant guidelines and regulations.

### Study population

We analysed data from the UK Biobank study that included more than 500,000 middle-aged (38–73 years) adults recruited from 22 sites across England, Wales, and Scotland. We used baseline data collected between 2006 and 2010^26,27^. Socio-demographic, lifestyle (smoking, alcohol consumption, dietary intake, physical activity, sitting time and sleep duration) and medical history were collected using the touchscreen questionnaire^27^.

### Disease categories

The UK Biobank collected self-reported medical information, such as CVD based on physician diagnosis. To define participants' CVD status, we used data on vascular/heart problems diagnosed by a doctor (Field ID = 6150). Under this Field ID, four CVDs were reported: heart attack, angina, stroke, and high blood pressure. For this analysis, participants who were reported to have at least one of these diseases were classified as having CVD, not otherwise. A total of 283,172 participants without missing data were included.

For the 174,030 participants without CVD, we computed a 10-year CVD risk score^28^ using the Framingham risk score function from the CVrisk package^29^ in R^30^. The variables included in the 10-year CVD risk calculation were age, gender, total cholesterol, HDL cholesterol, systolic blood pressure, blood pressure medication, smoking and diabetes status^28^.

### Lifestyle behaviours

This analysis used six lifestyle behaviours: smoking, physical activity, fruit and vegetable consumption, alcohol intake, screen and driving time, and sleep duration.

#### Physical activity

The UK Biobank collected data on physical activity using adapted questions from the short International Physical Activity Questionnaire (IPAQ)^31^ that includes the frequency, intensity and duration of walking, moderate and vigorous activity. UK Biobank data on time spent on moderate and vigorous activity was added and converted to a metabolic equivalent of task (MET) score. Participants were classified as active if they had ≥ 750 MET min/week or inactive (< 750 MET min/week), based on the 2019 AHA guideline^6^.

#### Fruit and vegetable intake

The UK Biobank collected data on dietary intake using the Food Frequency Questionnaire^32^. The NHS guideline recommends every individual to eat at least 5 portions of a variety of fruit and vegetables every day^33,34^. Data on fresh fruit (pieces), dried fruit (pieces), salad/raw vegetable (heaped tablespoons) and cooked vegetable (heaped tablespoons) were combined to calculate portions. Participants consuming at least 5 portions of fruits and vegetables per day were considered to have adequate fruit and vegetable intake.

#### Alcohol intake

Participants were asked for the number of pints of beer, glasses of wine, and measures of spirit consumed in the last week. Since alcoholic drinks differ in the amount of alcohol content, we converted each drink into equivalent standard units (1 unit contains 10 ml of ethyl alcohol)^35^. We calculated total weekly units of alcohol consumption by adding the units of beer, wine, and spirits. Based on the NHS guidelines^35^, we grouped participants as low-risk drinkers (≤ 14 units per week) or high-risk drinkers (> 14 units per week).

#### Smoking

To measure smoking status, participants were asked, "Do you smoke tobacco now?". Response options were “Yes, on most or all days”, “Only occasionally” and “No”. Those who responded “yes” or “smoke occasionally” were coded as 1, current smoker, while those who responded as “no” were coded as 0, not a current smoker.

#### Prolonged sitting

Total sitting time was calculated from the sum of self-reported hours spent watching television, using the computer, and driving during a typical day. Based on the estimated total sitting time, participants were categorized as low risk sitting (< 8 h/day) or prolonged sitting (≥ 8 h/day)^36,37^. This was based on the evidence of greater mortality risk for each increased sitting time category compared with < 8 h/day^36,37^.

#### Sleep duration

To measure sleep duration, the UK Biobank asked participants ‘About how many hours sleep do you get in every 24 h? (please include naps)’. Sleep duration was split using predefined thresholds from the literature; < 7 h, 7–8 h and > 8 h^13^. Based on these cut points, participants were grouped as having ‘poor sleep’ (< 7 or > 8 h/night) and ‘good sleep’ (7–8 h/night).

### Socio-demographic variables

Socio-demographic characteristics (age and gender), and Townsend deprivation index (TSDI) were included in the latent class analysis (LCA) model. TSDI was used to measure participants' deprivation^38^. The index combines information on housing, employment, car availability and social class, with higher values indicating greater deprivation^38^.

### Statistical analysis

**Figure 1:**
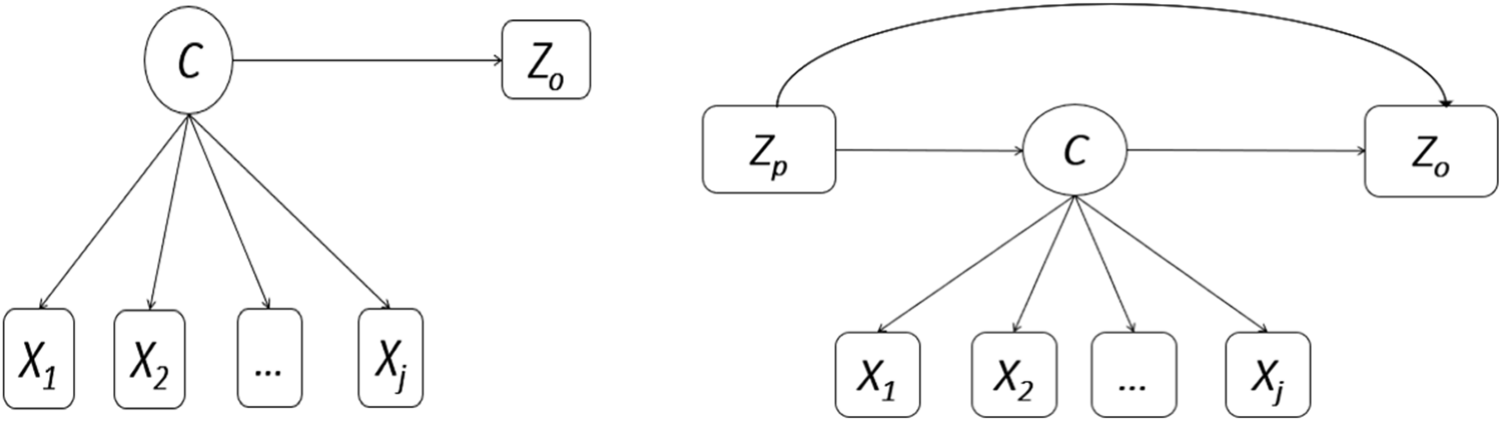
Graphical representation of latent class model with distal outcome and covariate. Where C = latent class, X_1_, X_2_, …, X_J_ refer to latent class indicators of C, Z_o_ = distal outcome, and Z_p_ = covariate of the latent class variable C and distal outcome Z_o_.

Descriptive statistics were performed on socio-demographic characteristics, lifestyle behaviours and medical conditions. The Mplus version 8.8 software^39^ was used to estimate a distal outcome model to identify latent classes (LCs) of lifestyle risk behaviours, and the association between LC membership and distal outcomes (CVD, and CVD risk) (Fig. 1). To select the number of LCs that best fit the data, we first fitted a two-class latent model and successively increased the number of LCs by one, up to a six-class latent model. Model evaluation was performed using the Bayesian Information Criterion (BIC) and Akaike information criterion (AIC)^40^. Model selection was made based on statistical criteria (with lower AIC and BIC) and interpretability of the estimated LCs^40^. Based on these criteria, four LCs for CVD, and three LCs for CVD risk were selected (see Supplementary Tables).

LC analysis with a distal outcome^22–24^, covariate, and LC mediator^41^ was run to identify and estimate: (1) the effect of LC membership on distal outcomes (Fig. 1—left hand side), and (2) the direct, indirect (LC membership mediated effect) and total effect of covariate(s) on distal outcomes (Fig. 1—right hand side). Gender, age, and smoking were not used in the CVD risk distal outcome model—since we used them in the computation of the CVD risk score. For distal outcome models, the Bolck–Croon–Hagenaars (BCH) method^22,42^ outperforms other methods. In addition, the BCH approach gives more accurate mediation estimates in LC analysis mediation models^41^. The BCH method avoids shifts in LC in the final step and performs well when the variance of the auxiliary variable differs substantially across classes^22^. To estimate the model, the BCH method uses weights that reflect the measurement error of the LC variable^22^. Two versions of the BCH method were implemented in Mplus—the automatic and two-step manual BCH versions^22^. The automatic BCH method is restrictive. In this analysis, we used the manual BCH two-step approach to estimate auxiliary models with continuous (CVD risk) and categorical (CVD) distal outcomes. We estimated the LC measurement model and saved the BCH weights in the first step. The second step estimated the general auxiliary model conditional on the LC variable using the BCH weights. Continuous (mean difference—MD) and binary (odds ratio—OR) outcomes were reported with 95% confidence intervals (95% CI).

## Results

### Population characteristics

**Figure 2:**
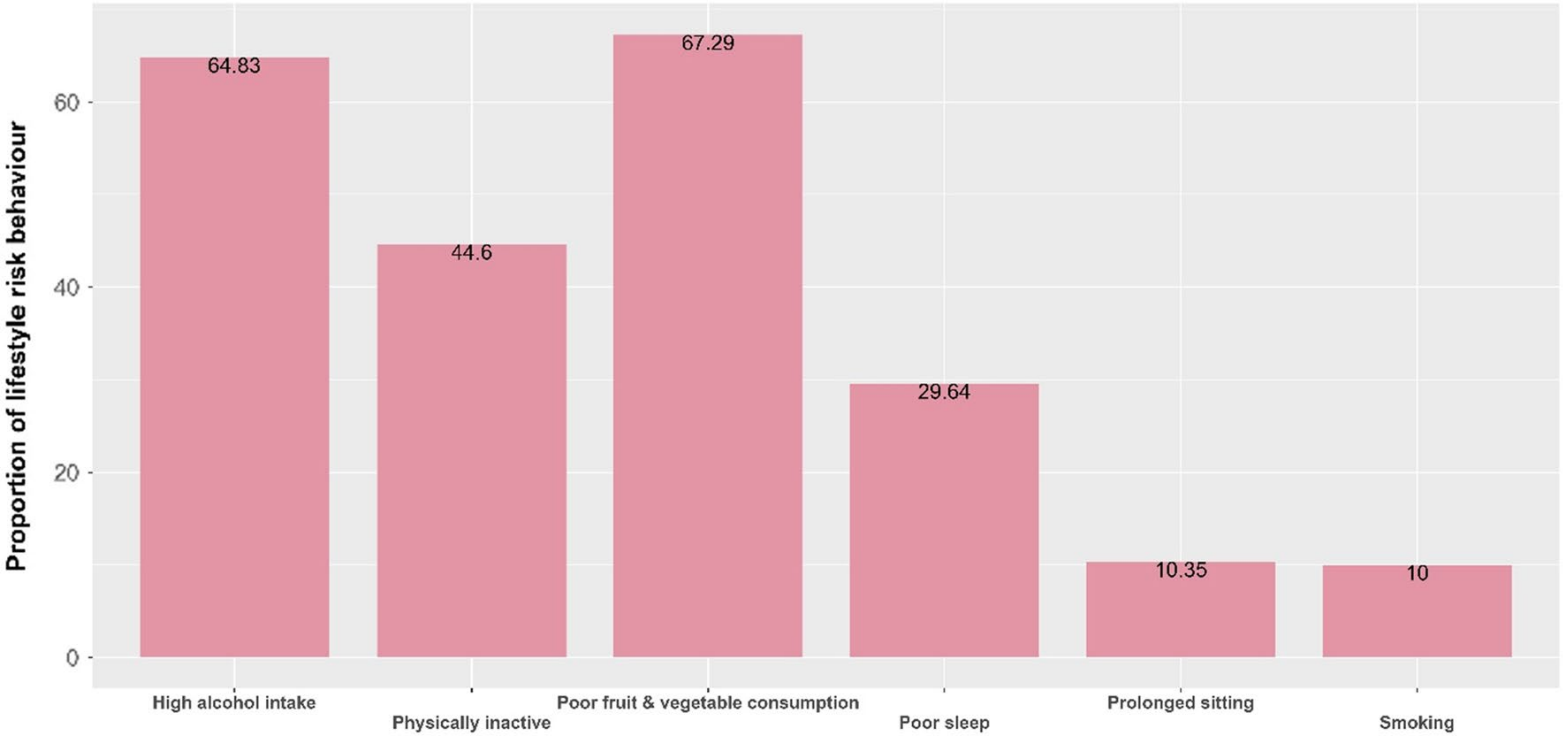
Lifestyle risk behaviour among UK adults with CVD data.

**Figure 3:**
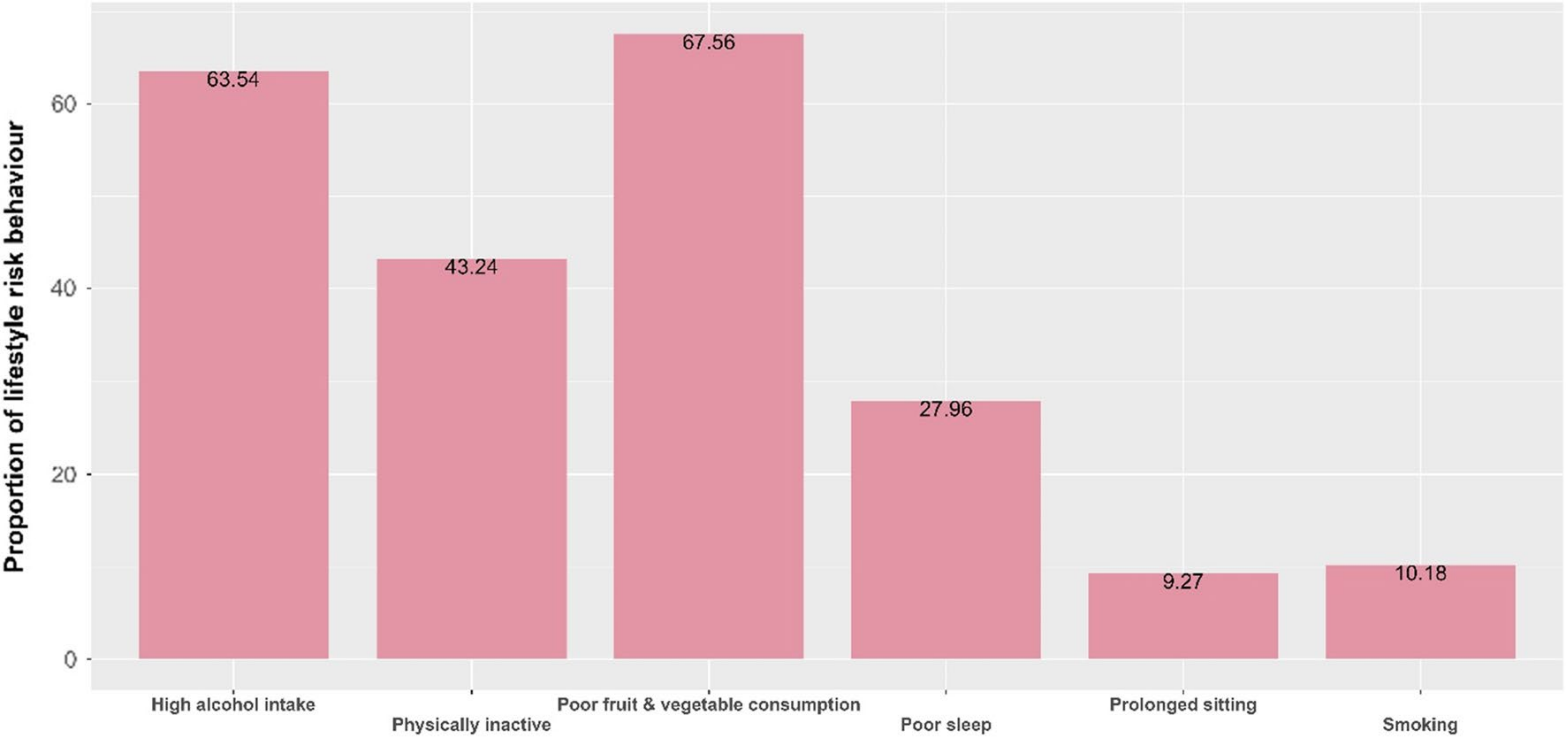
Lifestyle risk behaviours among UK adults with CVD risk.

We included UK adults who had data on CVD status (n = 283,172) and CVD risk (n = 174,030) along with lifestyle risk behaviours. The mean (SD) age of participants was 56.39 (8.02) and 55.15 (8.05) years for CVD and for those at risk of developing CVD, respectively. Among adults with CVD and at risk of developing CVD, 52.64% and 49.27% were males, respectively. Among adults with CVD, 67.29% had poor fruit and vegetable consumption, followed by high alcohol intake (64.83%) and were physically inactive (44.60%) (Fig. 2). Similarly, among adults at risk of developing CVD, 67.56% had poor fruit and vegetable intake and 63.54% had high alcohol intake (Fig. 3). Males, except for physical inactivity, had the highest proportion of lifestyle risk behaviours among those with CVD and at risk of developing CVD (see Supplementary Figs. S1, S2).

### Lifestyle risk behaviours

A model with three LCs were selected for adults at risk of developing CVD and four LCs for CVD outcome data. For adults at risk of developing CVD, LC 1 was characterised by physical inactivity (53.70%), poor fruit and vegetable intake (83.00%), high alcohol intake (74.60%), and prolonged sitting (100.00%). Adults in LC 2 had a high probability of high alcohol intake (58.40%). LC 3 had high probabilities of poor fruit and vegetable intake (100.00%) and high alcohol intake (65.00%) (Table 1).

**Table 1:** Lifestyle risk behaviour probabilities of UK adults at risk of developing CVD.

Variable	LC 1 (6.27%)	LC 2 (31.37%)	LC 3 (62.46%)
Physically inactive	**0.54**	0.34	0.47
Poor fruit and vegetable consumption	**0.83**	0.00	**1.00**
High alcohol intake	**0.75**	**0.58**	**0.65**
Poor sleep	0.38	0.28	0.27
Prolonged sitting	**1.00**	0.05	0.03

Probability values ≥ 0.50 are in bold.

For the CVD outcome, adults in the LC 1 had a high probability of being physically inactive (61.60%), poor fruit and vegetable consumption (83.70%) and high alcohol intake (77.50%). Participants in LC 2 had a high probability of high alcohol intake (64.80%). In LC 3, the probability of having poor fruit and vegetable intake and high alcohol intake were 91.60% and 80.60%, respectively. The LC 4 was characterised by physically inactive (52.00%) and poor fruit and vegetable intake (73.40%) (Table 2).

**Table 2:** Lifestyle risk behaviour probabilities of UK adults with CVD outcome data.

Variable	LC 1 (17.95%)	LC 2 (22.06%)	LC 3 (45.42%)	LC 4 (14.57%)
Physically inactive	**0.62**	0.29	0.43	**0.52**
Poor fruit and vegetable consumption	**0.84**	0.00	**0.92**	**0.73**
High alcohol intake	**0.78**	**0.65**	**0.81**	0.00
Poor sleep	0.46	0.29	0.24	0.27
Smoking	0.23	0.05	0.11	0.00
Prolonged sitting	0.29	0.07	0.06	0.05

Probability values ≥ 0.50 are in bold.

### Effects of latent class membership on CVD risk

LC membership had a statistically significant effect on CVD risk. Being in LC 1 (physically inactive, poor fruit & vegetable intake, high alcohol intake, and prolonged sitting) was associated with increased CVD risk compared with LC 2 (MD = 3.29 [3.12, 3.46]). Similarly, adults in LC 3 (poor fruit & vegetable intake, and high alcohol intake) had a 0.89 mean increased CVD risk relative to LC 2 (Table 3). Similarly, social deprivation measured in TSDI, except for total effect, showed a statistically significant effect on CVD risk (Table 3).

**Table 3:** Latent class membership and TSDI associated with CVD risk: multivariable analyses (mean difference and 95% confidence intervals).

Variables	MD (95% CI)
**LC membership on CVD risk**	
LC 1: physically inactive, poor fruit & vegetable intake, high alcohol intake, and prolonged sitting	**3.29 (3.12, 3.46)**
LC 2: high alcohol intake	Reference
LC 3: poor fruit and vegetable intake, and high alcohol intake	**0.89 (0.82, 0.96)**
**TSDI effect on CVD risk**	
Direct effect	**− 0.02 (− 0.03, − 0.01)**
Indirect (LC membership mediated effect) effect	**0.01 (0.01, 0.01)**
Total effect	− 0.01 (− 0.02, 0.00)

Significant values are in bold.

### Effects of latent class membership on CVD status

The odds of developing CVD were significantly associated with an individual’s LC membership. UK adults who belonged to LC 1 (Physically inactive, poor fruit and vegetable intake, and high alcohol intake) had 25.18 higher odds of having CVD than those in LC 4 (Physically inactive, and poor fruit and vegetable intake). Similarly, compared to those in LC 4, the odds of having CVD was higher among those who were in LC 2 (7.70) and LC 3 (5.19). In addition, gender, age and TSDI showed statistically significant effects on the odds of developing CVD. The direct, indirect, and total effects of being male were 1.19, 1.37- and 1.63-times higher odds of having CVD than females, respectively. A single-year increase in the age of adults, except for the indirect effect, was significantly associated with higher odds of developing CVD. For UK adults, a one-point increase in the TSDI score was associated with higher odds of having CVD (Table 4).

**Table 4:** Latent class membership and other factors associated with CVD: multivariable analyses (odds ratios and 95% confidence intervals).

Variables	OR (95% CI)
**LC membership on CVD status**	
LC 1: physically inactive, poor fruit and vegetable intake, and high alcohol intake	**25.18 (7.02, 43.33)**
LC 2: high alcohol intake	**7.70 (2.31, 13.08)**
LC 3: poor fruit and vegetable intake, and high alcohol intake	**5.19 (1.55, 8.82)**
LC 4: physically inactive, and poor fruit and vegetable intake	Reference
**Direct effect of covariates on CVD status**	
Male	**1.19 (1.04, 1.34)**
Age	**1.08 (1.07, 1.09)**
TSDI	**1.02 (1.01, 1.03)**
**Indirect (LC membership mediated effect) effect of covariates on CVD status**	
Male	**1.37 (1.24, 1.50)**
Age	0.99 (0.99, 1.00)
TSDI	**1.08 (1.06, 1.10)**
**Total effect of covariates on CVD status**	
Male	**1.63 (1.36, 1.91)**
Age	**1.07 (1.06, 1.08)**
TSDI	**1.11 (1.08, 1.13)**

Significant values are in bold.

## Discussion

In this study, we demonstrated that clustering of multiple lifestyle risk behaviours in adults significantly increased the risk of CVD and being diagnosed with CVD. Individuals’ latent class membership with two or more lifestyle risk behaviours were significantly associated with CVD risk and being diagnosed with CVD. Socioeconomic characteristics were also associated with being diagnosed with CVD.

The likelihood of developing CVD within a given time depends on the number of risk factors. Individuals' latent class membership with multiple lifestyle risk behaviours showed a 3.29 mean increase in the risk of developing CVD relative to a single risky behaviour. Smoking, physical inactivity, alcohol use, and low fruit and vegetable intake had the highest effect on NCD development and death^8^. On the other hand, lifestyle modification can reduce individuals’ risk of developing CVD. Adherence to Life's Simple 7 metrics has been associated with a lower rate of cardiovascular events^43^. In a meta-analysis of prospective cohort studies on the effect of a combined healthy lifestyle (healthy diet, moderate alcohol consumption, non-smoking, physical activity, and optimal weight) on CVD risk, people with the highest number of healthy lifestyle factors had lower CVD risk relative to those with the lowest number of healthy lifestyle factors [pooled hazard ratio = 0.37 (95% CI 0.31–0.43)]^44^.

Clustering of two or more lifestyle risk behaviours could have a synergetic effect on CVD. Adults with clustering of three lifestyle risk behaviours (physically inactive, poor fruit and vegetable intake, and high alcohol intake) had 25.18 higher odds of having CVD than those with two lifestyle risk behaviours (physically inactive, and poor fruit and vegetable intake). In a systematic review of longitudinal observational studies, the combination of physical inactivity with smoking, high alcohol intake, poor diet, or sedentary behaviour showed increased CVD incidents, death due to CVD and/or any other cause^45^. However, smoking, and prolonged sitting did not show significant contributions to the LC membership and CVD status. It could be due to the small proportion, which needs further investigation.

Lifestyle risk behaviours are also associated with an increased risk of premature mortality. In a meta-analysis, years-of-life-lost due to high alcohol intake was 0.5 years, 2.4 years for physical inactivity, and 4·8 years for smoking^46^. In addition, the combination of multiple lifestyle risk behaviours showed increased risks of all-cause and/or cardiometabolic mortalities^47,48^. The co-occurrence of smoking, physical inactivity and poor social participation increased cardiometabolic mortality by 3.13 relative to no-risk behaviour^47^. On the contrary, meeting the cardiovascular health metrics and engaging in a healthy lifestyle were associated with lower incidences of CVD, cardiovascular mortality, and all-cause mortality^49,50^. Therefore, more emphasis should be placed on interventions targeting multiple lifestyle behaviours.

In addition, adults with increasing socioeconomic deprivation scores had an increased risk of being diagnosed with CVD. People living in socioeconomic deprived areas had multiple lifestyle risk behaviours, experience high CVD rates, and premature mortality^46,51,52^. In a study conducted in the UK, areas with higher socioeconomic deprivation had high coronary heart disease mortality^53^. Populations with socioeconomic deprivation are more likely to smoke, have a poor diet, and not exercise enough^52^. To reduce the effect of social deprivation on health, effective policies and strategies should be designed to modify socioeconomic circumstances and their consequences.

The odds of being diagnosed with CVD varied according to sex and age. In most cases, male and a single-year increase in the age of adults were significantly associated with the odds of being diagnosed with CVD. In a study conducted on gender-specific, lifestyle-related behaviours and 10-year CVD risk, males had higher CVD events—both first and recurrent CVD relative to females^54^. Regarding the effect of age, a meta-analysis reported that the hazard ratio of CVD increased with increasing age of adults^44^. Younger adults have more cardiovascular benefits from the combined effects of a healthy lifestyle^44,50^. Therefore, lifestyle interventions targeted towards younger people are needed to prevent CVD.

Our study has several strengths. LCA, a cross-sectional latent variable mixture modelling, has several advantages over other traditional methods used in lifestyle risk behaviour^55,56^. First, it uses maximum likelihood estimation to identify subgroups that are internally homogenous and externally heterogeneous^57^. Second, it is a model-based technique, which has an advantage over heuristic cluster techniques (e.g., k-means clustering) in that it provides fit statistics^40^. Fit statistics are useful in selecting the most appropriate model for the data^58^ and to compare models for hypothesis testing^59^. Third, LCA provides information on the probability that an individual is within a particular class^58^. This has significant importance for researchers and practitioners in the field to identify subgroups of lifestyle risk behaviours and a targeted approach to healthy lifestyle promotion. These subgroups can be studied further to investigate problems related to lifestyle risk behaviour classes that are commonly found in the general population, how prevalent they are, what causes them, what future outcomes they predict (distal outcomes), and whether lifestyle risk behaviour classes change over time.

Despite these strengths, our findings should be considered with the following limitations in mind. First, lifestyle risk behaviours were measured based on self-reported questionnaires, which could have recall bias or social desirability bias. Second, the measures of several lifestyle risk behaviours are under-specified; for example, the sleep measure was limited quantity only, without considering sleep quality. The smoking measure also did not consider past smoking.

## Conclusion

Latent class membership with two or more lifestyle risk behaviours showed an increased risk of developing CVD and CVD events due to the potential synergetic relationships among lifestyle risk factors. Deprived populations are more likely to be affected by CVD from the wide combination of lifestyle risk behaviours. Early interventions targeting multiple lifestyle risk behaviours from young age should be prioritized to prevent future CVD events.

## Supplementary Information

Supplementary Information.

## Data Availability

All data relevant to the study are included in the article.
