# Supplementary material for: Effects of lifestyle risk behaviour clustering on cardiovascular disease among UK adults: latent class analysis with distal outcomes
Source: Sci Rep. 2022 Oct 17;12:17349. doi: 10.1038/s41598-022-22469-6 (PMC9576714; doi:10.1038/s41598-022-22469-6)
Supplement: Supplementary file 1 — Supplementary Information. [file 41598_2022_22469_MOESM1_ESM.docx]

Supplementary Information belonging to:

**Effects of lifestyle risk behaviour clustering on cardiovascular disease among UK adults: latent class analysis with distal outcomes**

Teketo Kassaw Tegegne, Shariful Islam, Ralph Maddison

The selected latent classes are highlighted in pink rectangle based on model fit statistics and interpretability of each latent class. A 50% or higher classification probabilities cut-off was considered for the most likely latent class membership in which each latent class describes a distinct and meaningful pattern.

**Supplementary Information for CVD at risk data**

**Supplementary Table S1: Model fit summary statistics: CVD risk data**

| **Latent class (LC)** | **AIC** | **BIC** |
| --- | --- | --- |
| LC 1 | 999473.260 | 999523.595 |
| LC 2 | 995927.440 | 996038.176 |
| LC 3 | 994994.211 | 995165.350 |
| **LC 4** | **994726.650** | **994958.190** |
| LC 5 | 994710.971 | 995002.914 |

**Supplementary Table S2: Latent class 1: Lifestyle risk behaviour probabilities of UK adults at risk of developing CVD**

| **Variable** | **LC 1** |
| --- | --- |
| Physically inactive | 0.432 |
| Poor fruit & vegetable consumption | **0.676** |
| High alcohol intake | **0.635** |
| Poor sleep | 0.280 |
| Prolonged sitting | 0.093 |

**Supplementary Table S3: Latent class 2: Lifestyle risk behaviour probabilities of UK adults at risk of developing CVD**

| **Variable** | **LC 1** | **LC 2** |
| --- | --- | --- |
| Physically inactive | 0.474 | 0.346 |
| Poor fruit & vegetable consumption | **1.000** | 0.000 |
| High alcohol intake | **0.658** | **0.589** |
| Poor sleep | 0.279 | 0.281 |
| Prolonged sitting | 0.100 | 0.077 |

**Supplementary Table S4: Latent class 3: Lifestyle risk behaviour probabilities of UK adults at risk of developing CVD**

| **Variable** | **LC 1** | **LC 2** | **LC 3** |
| --- | --- | --- | --- |
| Physically inactive | **0.537** | 0.341 | 0.468 |
| Poor fruit & vegetable consumption | **0.830** | 0.000 | **1.000** |
| High alcohol intake | **0.746** | **0.584** | **0.650** |
| Poor sleep | 0.378 | 0.277 | 0.271 |
| Prolonged sitting | **1.000** | 0.046 | 0.025 |

**Supplementary Table S5: Latent class 4: Lifestyle risk behaviour probabilities of UK adults at risk of developing CVD**

| **Variable** | **LC 1** | **LC 2** | **LC 3** | **LC 4** |
| --- | --- | --- | --- | --- |
| Physically inactive | 0.322 | 0.000 | 0.486 | **0.578** |
| Poor fruit & vegetable consumption | 0.000 | **0.546** | **0.868** | **0.815** |
| High alcohol intake | 0.478 | **0.910** | **0.604** | **0.723** |
| Poor sleep | 0.275 | 0.273 | 0.243 | 0.376 |
| Prolonged sitting | 0.043 | 0.093 | 0.018 | 0.315 |

**Supplementary Table S6: Latent class 5: Lifestyle risk behaviour probabilities of UK adults at risk of developing CVD**

| **Variable** | **LC 1** | **LC 2** | **LC 3** | **LC 4** | **LC 5** |
| --- | --- | --- | --- | --- | --- |
| Physically inactive | 0.365 | **0.585** | 0.197 | 0.000 | 0.490 |
| Poor fruit & vegetable consumption | 0.117 | **0.835** | 0.000 | **0.509** | **0.805** |
| High alcohol intake | 0.447 | **0.735** | 0.375 | **0.917** | **0.604** |
| Poor sleep | 0.374 | 0.374 | 0.165 | 0.275 | 0.242 |
| Prolonged sitting | 0.097 | 0.317 | 0.000 | 0.089 | 0.021 |

**Supplementary Information for CVD outcome data**

**Supplementary Table S7: Model fit summary statistics: CVD outcome data**

| **Latent class (LC)** | **AIC** | **BIC** |
| --- | --- | --- |
| LC 1 | 1831277.803 | 1831341.126 |
| LC 2 | 1821668.248 | 1821805.448 |
| LC 3 | 1818658.041 | 1818869.118 |
| LC 4 | 1818009.643 | 1818294.596 |
| **LC 5** | **1817716.960** | **1818149.666** |
| LC 6 | 1817803.478 | 1818162.307 |

**Supplementary Table S8: Latent class 1: Lifestyle risk behaviour probabilities of UK adults with CVD outcome data**

| **Variable** | **LC 1** |
| --- | --- |
| Physically inactive | 0.446 |
| Poor fruit & vegetable consumption | **0.673** |
| High alcohol intake | **0.648** |
| Poor sleep | 0.296 |
| Smoking | 0.100 |
| Prolonged sitting | 0.104 |

**Supplementary Table S9: Latent class 2: Lifestyle risk behaviour probabilities of UK adults with CVD outcome data**

| **Variable** | **LC 1** | **LC 2** |
| --- | --- | --- |
| Physically inactive | **0.573** | 0.367 |
| Poor fruit & vegetable consumption | **0.873** | **0.549** |
| High alcohol intake | **0.772** | **0.571** |
| Poor sleep | 0.342 | 0.268 |
| Smoking | 0.205 | 0.035 |
| Prolonged sitting | 0.169 | 0.063 |

**Supplementary Table S10: Latent class 3: Lifestyle risk behaviour probabilities of UK adults with CVD outcome data**

| **Variable** | **LC 1** | **LC 2** | **LC 3** |
| --- | --- | --- | --- |
| Physically inactive | **1.000** | **0.543** | 0.000 |
| Poor fruit & vegetable consumption | **0.668** | **0.851** | **0.551** |
| High alcohol intake | **0.522** | **0.846** | **0.594** |
| Poor sleep | 0.279 | 0.355 | 0.267 |
| Smoking | 0.027 | 0.256 | 0.039 |
| Prolonged sitting | 0.082 | 0.183 | 0.062 |

**Supplementary Table S11: Latent class 4: Lifestyle risk behaviour probabilities of UK adults with CVD outcome data**

| **Variable** | **LC 1** | **LC 2** | **LC 3** | **LC 4** |
| --- | --- | --- | --- | --- |
| Physically inactive | **0.616** | 0.290 | 0.431 | **0.520** |
| Poor fruit & vegetable consumption | **0.837** | 0.000 | **0.916** | **0.734** |
| High alcohol intake | **0.775** | **0.648** | **0. 806** | 0.000 |
| Poor sleep | 0.462 | 0.286 | 0.244 | 0.269 |
| Smoking | 0.225 | 0.047 | 0.108 | 0.000 |
| Prolonged sitting | 0.292 | 0.072 | 0.062 | 0.049 |

**Supplementary Table S12: Latent class 5: Lifestyle risk behaviour probabilities of UK adults with CVD outcome data**

| **Variable** | **LC 1** | **LC 2** | **LC 3** | **LC 4** | **LC 5** |
| --- | --- | --- | --- | --- | --- |
| Physically inactive | **0.648** | 0.362 | 0.250 | **0.537** | **0.564** |
| Poor fruit & vegetable consumption | **0.785** | 0.311 | **0.573** | **1.000** | **0.970** |
| High alcohol intake | **0.730** | 0.455 | **1.000** | **0.590** | **0.888** |
| Poor sleep | 0.464 | 0.282 | 0.270 | 0.247 | 0.379 |
| Smoking | 0.139 | 0.035 | 0.111 | 0.052 | **0.733** |
| Prolonged sitting | 0.367 | 0.053 | 0.091 | 0.050 | 0.161 |

**Supplementary Table S13: Latent class 6: Lifestyle risk behaviour probabilities of UK adults with CVD outcome data**

| **Variable** | **LC 1** | **LC 2** | **LC 3** | **LC 4** | **LC 5** | **LC 6** |
| --- | --- | --- | --- | --- | --- | --- |
| Physically inactive | **0.553** | 0.095 | **0.656** | 0.461 | **0.648** | 0.224 |
| Poor fruit & vegetable consumption | **0.878** | **0.568** | **0.769** | 0.451 | **0.889** | 0.473 |
| High alcohol intake | **0.800** | **1.000** | **0.644** | **0.573** | **0.822** | 0.000 |
| Poor sleep | 0.326 | 0.257 | 0.267 | **0.545** | 0.458 | 0.249 |
| Smoking | **1.000** | 0.065 | 0.010 | 0.067 | 0.295 | 0.031 |
| Prolonged sitting | 0.053 | 0.066 | 0.074 | 0.223 | 0.488 | 0.036 |

**Supplementary Figure S1: Lifestyle risk behaviour among UK adults with CVD data by gender**


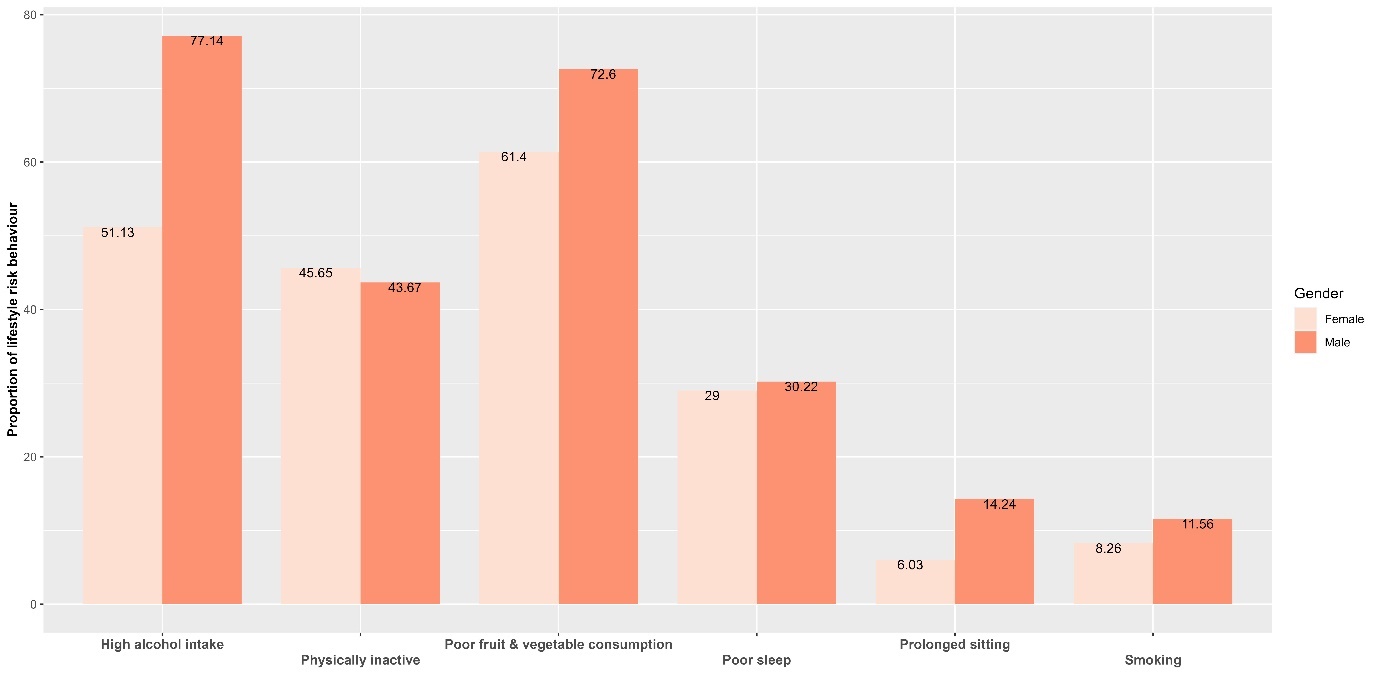


**Supplementary Figure S2: Lifestyle risk behaviours among UK adults with CVD risk by gender**


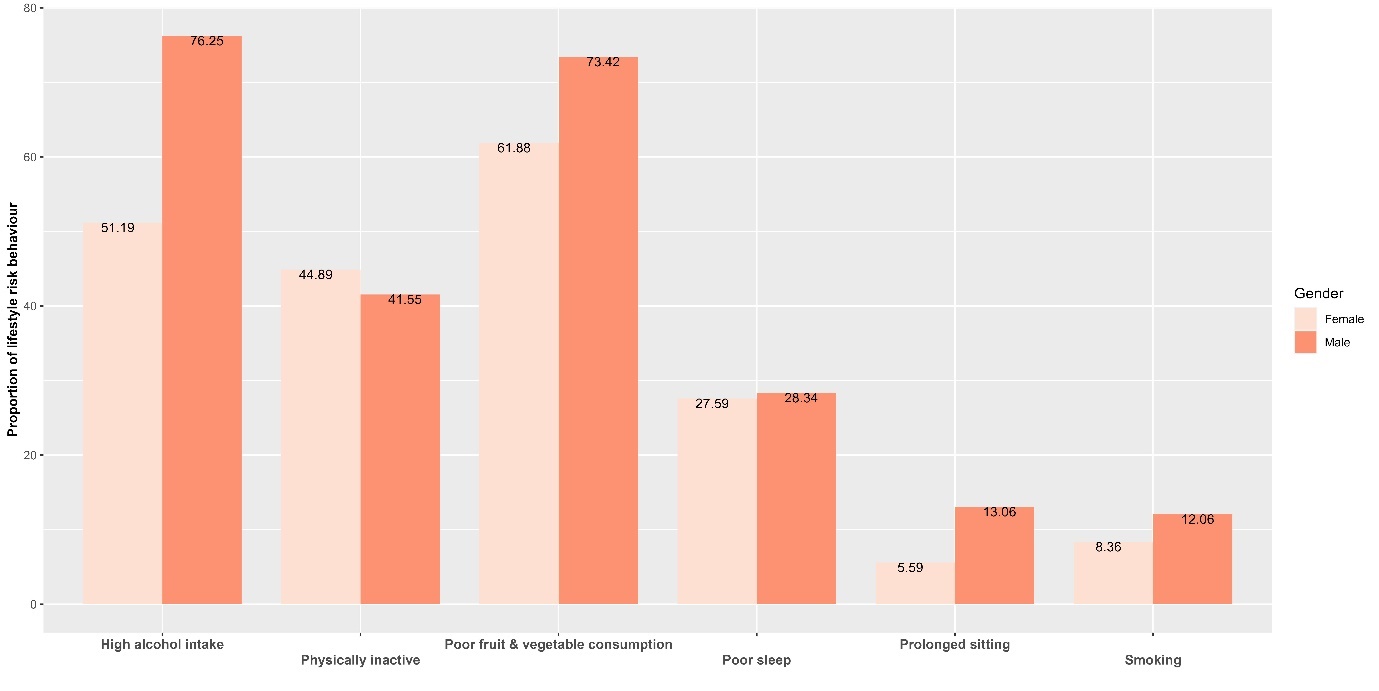


**Supplementary Mplus Code S1: CVD risk**

**STEP 1 BCH CODE: CVD RISK**

TITLE:

Step one BCH estimation - LCA with distal outcome (cvd risk).

DATA:

file = risk_score.csv;

VARIABLE:

names = id tsdi pa veg alcohol sleep seden frs;

classes = c(3);

usevar = pa-seden;

categorical = pa-seden;

idvariable = id;

auxiliary = tsdi frs;

ANALYSIS:

type=mixture;

estimator = mlr;

starts = 200 100;

processors = 8;

MODEL:

%overall%

%C#1%

[pa$1-seden$1];

%C#2%

[pa$1-seden$1];

%C#3%

[pa$1-seden$1];

SAVEDATA:

file = risk.txt;

save = bch;

**STEP 2 BCH CODE: CVD RISK**

title:

Nominal C, Continuous Y (where Y is FRS)

Using a latent class variable to represent C

Simulating x-c interaction effect on y by class-varying y on x (where X is TSDI)

Step 2: BCH approach on LCA with distal outcome - CVd Risk

DATA:

file=risk.txt;

! Predictor variable - X (TSDI)

! Mediator variable(s) – C

! Distal outcome variable - Y (FRS)

VARIABLE:

names = PA VEG ALC SL SED X Y

BCHW1 BCHW2 BCHW3 ID;

classes = c(3);

usevar = X Y BCHW1 BCHW2 BCHW3;

training = BCHW1-BCHW3(bch);

ANALYSIS:

type = mixture;

estimator = mlr;

processors = 8;

starts = 0;

MODEL:

%overall%

[c#1] (gamma01);

[c#2] (gamma02);

c#1 on x (gamma11);

c#2 on x (gamma12);

y on x; !direct effect

y; ! estimating variance

%c#1%

[y] (beta01); ! This is to estimate class-specific mean for y,

! and we coded this ‘beta01’ in parentheses for later use in the MODEL CONSTRAINT.

y on x (beta11); ! This is to estimate the effect of covariate x on the outcome variable y,

! and we coded this ‘beta11’ in parentheses for later use in the MODEL CONSTRAINT.

%c#2%

[y] (beta02); ! class-specific mean for y, coded ‘beta02’

y on x (beta12); ! effect of covariate x on the outcome variable y, coded ‘beta12’

%c#3%

[y] (beta03); ! class-specific mean for y, coded ‘beta03’

y on x (beta13); ! effect of covariate x on the outcome variable y, coded ‘beta13’

MODEL CONSTRAINT:

new(denom0 denom1 p10 p11 p20

p21 p30 p31 term11 term10

term01 term00

de tie total pie

m12 m32 !mean difference - LC membership effect on CVD risk

);

! index is x' for multinomial denominator

denom0=exp(gamma01)+exp(gamma02)+1;

denom1=exp(gamma01+gamma11)+exp(gamma02+gamma12)+1;

! first index is class, second x' for probabilities

p10=exp(gamma01)/denom0;

p11=exp(gamma01+gamma11)/denom1;

p20=exp(gamma02)/denom0;

p21=exp(gamma02+gamma12)/denom1;

p30=1/denom0;

p31=1/denom1;

! first index is x, second x', summing over class

term11=(beta01+beta11)*p11+(beta02+beta12)*p21+(beta03+beta13)*p31;

term10=(beta01+beta11)*p10+(beta02+beta12)*p20+(beta03+beta13)*p30;

term01=(beta01)*p11+(beta02)*p21+(beta03)*p31;

term00=(beta01)*p10+(beta02)*p20+(beta03)*p30;

de=term10-term00;

tie=term11-term10;

total=term11-term00;

pie=term01-term00;

m12 = beta01 - beta02;

!compute mean difference between m1 and m2 WHERE m2 (ALCOHOL ONLY) is REFERENCE

m32 = beta03 - beta02;

!compute mean difference between m3 and m2 WHERE m2 (ALCOHOL ONLY) is REFERENCE

OUTPUT:

CINTERVAL;

**Supplementary Mplus Code S2: CVD outcome**

**STEP 1 BCH CODE: CVD OUTCOME**

TITLE:

Step one of the BCH LCA with distal outcome analysis on lifestyle risk behviour.

DATA:

file = cvd_2.csv;

VARIABLE:

names = id gender age tsdi pa veg alcohol sleep smoke seden cvd;

classes = c(4);

usevar = pa-seden;

categorical = pa-seden;

idvariable = id;

auxiliary = gender age tsdi cvd;

ANALYSIS:

type=mixture;

estimator = mlr;

starts = 100 25;

MODEL:

%overall%

%C#1%

[pa$1-seden$1];

%C#2%

[pa$1-seden$1];

%C#3%

[pa$1-seden$1];

%C#4%

[pa$1-seden$1];

SAVEDATA:

file = cvd_step1.txt; save = bch;

**STEP 2 BCH CODE: CVD OUTCOME**

TITLE:

Step two of the BCH LCA with distal outcome analysis on lifestyle risk behviour.

DATA:

file = cvd_step1.txt;

VARIABLE:

names = PA VEG ALCOHOL SLEEP SMOKE SEDEN GENDER AGE TSDI CVD

BCHW1 BCHW2 BCHW3 BCHW4 ID;

classes = c(4);

usevar = GENDER AGE TSDI CVD BCHW1 BCHW2 BCHW3 BCHW4;

categorical = CVD;

training = BCHW1-BCHW4(bch);

DEFINE:

center gender (grandmean);

ANALYSIS:

type = mixture;

starts = 0;

estimator = mlr;

processors = 8;

MODEL:

%overall%

[c#1] (gamma01);

[c#2] (gamma02);

[c#3] (gamma03);

c#1 ON gender (gamma11);

c#1 ON age (gamma21);

c#1 ON tsdi (gamma31);

c#2 ON gender (gamma12);

c#2 ON age (gamma22);

c#2 ON tsdi (gamma32);

c#3 ON gender (gamma13);

c#3 ON age (gamma23);

c#3 ON tsdi (gamma33);

cvd ON gender age tsdi ;

%c#1%

[cvd$1] (beta01);

cvd on gender (beta11);

cvd on age (beta21);

cvd on tsdi (beta31);

%c#2%

[cvd$1] (beta02);

cvd on gender (beta12);

cvd on age (beta22);

cvd on tsdi (beta32);

%c#3%

[cvd$1] (beta03);

cvd on gender (beta13);

cvd on age (beta23);

cvd on tsdi (beta33);

%c#4%

[cvd$1] (beta04);

cvd on gender (beta14);

cvd on age (beta24);

cvd on tsdi (beta34);

MODEL CONSTRAINT:

new(denom0 denom1 denom2 denom3

p11_gender p11_age p11_tsdi

p21_gender p21_age p21_tsdi

p31_gender p31_age p31_tsdi

p41_gender p41_age p41_tsdi

p10 p20 p30 p40

term11_gender term11_age term11_tsdi

term10_gender term10_age term10_tsdi

term01_gender term01_age term01_tsdi

term00

pnde_gender pnde_age pnde_tsdi

tnie_gender tnie_age tnie_tsdi

total_gender total_age total_tsdi

pnie_gender pnie_age pnie_tsdi

orpnde_gender orpnde_age orpnde_tsdi

ortnie_gender ortnie_age ortnie_tsdi

orpnie_gender orpnie_age orpnie_tsdi

ortotal_gender ortotal_age ortotal_tsdi !odds ratio - total effect

p1 p2 p3 p4 !probabilities

odds1 odds2 odds3 odds4 !odds

or12 or32 or42 !odds ratio ! ref = class 2

or13 or23 or43 ! ref = class 3

or14 or24 or34 ! ref = class 4

diff12 diff32 diff42);

! mediator probabilities:

! index is x0 for multinomial denominator

denom0 = exp(gamma01) + exp(gamma02) + exp(gamma03) + 1;

denom1 = exp(gamma01 + gamma11) + exp(gamma02 + gamma12) +

exp(gamma03 + gamma13) + 1; !for gender

denom2 = exp(gamma01 + gamma21) + exp(gamma02 + gamma22)

+ exp(gamma03 + gamma23) + 1; !for age

denom3 = exp(gamma01 + gamma31) + exp(gamma02 + gamma32)

+ exp(gamma03 + gamma33) + 1; !for tsdi

! first index is class, second x0 for probabilities

p10 = exp(gamma01)/denom0;

p11_gender = exp(gamma01 + gamma11)/denom1;

p11_age = exp(gamma01 + gamma21)/denom2;

p11_tsdi = exp(gamma01 + gamma31)/denom3;

p20 = exp(gamma02)/denom0;

p21_gender = exp(gamma02 + gamma12)/denom1;

p21_age = exp(gamma02 + gamma22)/denom2;

p21_tsdi = exp(gamma02 + gamma32)/denom3;

p30 = exp(gamma03)/denom0;

p31_gender = exp(gamma03 + gamma13)/denom1;

p31_age = exp(gamma03 + gamma23)/denom2;

p31_tsdi = exp(gamma03 + gamma33)/denom3;

p40 = 1/denom0;

p41_gender = 1/denom1;

p41_age = 1/denom2;

p41_tsdi = 1/denom3;

! outcome probabilities:

! first index is x1, second x0, summing over class

term11_gender = (1/(1+exp(beta01-beta11)))*p11_gender +

(1/(1+exp(beta02-beta12)))*p21_gender +

(1/(1+exp(beta03-beta13)))*p31_gender +

(1/(1+exp(beta04-beta14)))*p41_gender;

term11_age = (1/(1+exp(beta01-beta21)))*p11_age +

(1/(1+exp(beta02-beta22)))*p21_age +

(1/(1+exp(beta03-beta23)))*p31_age +

(1/(1+exp(beta04-beta24)))*p41_age;

term11_tsdi = (1/(1+exp(beta01-beta31)))*p11_tsdi +

(1/(1+exp(beta02-beta32)))*p21_tsdi +

(1/(1+exp(beta03-beta33)))*p31_tsdi +

(1/(1+exp(beta04-beta34)))*p41_tsdi;

term10_gender = (1/(1+exp(beta01-beta11)))*p10 +

(1/(1+exp(beta02-beta12)))*p20 +

(1/(1+exp(beta03-beta13)))*p30 +

(1/(1+exp(beta04-beta14)))*p40;

term10_age = (1/(1+exp(beta01-beta21)))*p10 +

(1/(1+exp(beta02-beta22)))*p20 +

(1/(1+exp(beta03-beta23)))*p30 +

(1/(1+exp(beta04-beta24)))*p40;

term10_tsdi = (1/(1+exp(beta01-beta31)))*p10 +

(1/(1+exp(beta02-beta32)))*p20 +

(1/(1+exp(beta03-beta33)))*p30 +

(1/(1+exp(beta04-beta34)))*p40;

term01_gender = (1/(1+exp(beta01)))*p11_gender +

(1/(1+exp(beta02)))*p21_gender +

(1/(1+exp(beta03)))*p31_gender +

(1/(1+exp(beta04)))*p41_gender;

term01_age = (1/(1+exp(beta01)))*p11_age +

(1/(1+exp(beta02)))*p21_age +

(1/(1+exp(beta03)))*p31_age +

(1/(1+exp(beta04)))*p41_age;

term01_tsdi = (1/(1+exp(beta01)))*p11_tsdi +

(1/(1+exp(beta02)))*p21_tsdi +

(1/(1+exp(beta03)))*p31_tsdi +

(1/(1+exp(beta04)))*p41_tsdi;

term00 = (1/(1+exp(beta01)))*p10+(1/(1+exp(beta02)))*p20+

(1/(1+exp(beta03)))*p30 +(1/(1+exp(beta04)))*p40;

! effects:

!pure natural direct effect (PNDE):

pnde_gender = term10_gender - term00;

pnde_age = term10_age - term00;

pnde_tsdi = term10_tsdi - term00;

!total natural indirect effect (TNIE)

tnie_gender = term11_gender - term10_gender;

tnie_age = term11_age - term10_age;

tnie_tsdi = term11_tsdi - term10_tsdi;

total_gender = term11_gender - term00;

total_age = term11_age - term00;

total_tsdi = term11_tsdi - term00;

!pure natural indirect effect (PNIE)

pnie_gender = term01_gender - term00;

pnie_age = term01_age - term00;

pnie_tsdi = term01_tsdi - term00;

!pure natural direct effect (PNDE): odds ratio

orpnde_gender = (term10_gender/(1-term10_gender))/(term00/(1-term00));

orpnde_age = (term10_age/(1-term10_age))/(term00/(1-term00));

orpnde_tsdi = (term10_tsdi/(1-term10_tsdi))/(term00/(1-term00));

!total natural indirect effect (TNIE): odds ratio

ortnie_gender = (term11_gender/(1-term11_gender))/(term10_gender/(1-term10_gender));

ortnie_age = (term11_age/(1-term11_age))/(term10_age/(1-term10_age));

ortnie_tsdi = (term11_tsdi/(1-term11_tsdi))/(term10_tsdi/(1-term10_tsdi));

!pure natural indirect effect (PNIE): odds ratio

orpnie_gender = (term01_gender/(1-term01_gender))/(term00/(1-term00));

orpnie_age = (term01_age/(1-term01_age))/(term00/(1-term00));

orpnie_tsdi = (term01_tsdi/(1-term01_tsdi))/(term00/(1-term00));

!total effect: odds ratio

ortotal_gender = (term11_gender/(1-term11_gender))/(term00/(1-term00));

ortotal_age = (term11_age/(1-term11_age))/(term00/(1-term00));

ortotal_tsdi = (term11_tsdi/(1-term11_tsdi))/(term00/(1-term00));

!compute the probability at age, gender and tsdi level zero

!gender = 0

!age = 0

!tsdi = 0

p1 = 1/(1 + exp(beta01)); !t1 is replaced by beta01 - LC1

p2 = 1/(1 + exp(beta02)); !t2 is replaced by beta02 - LC2

p3 = 1/(1 + exp(beta03)); !t3 is replaced by beta03 - LC3

p4 = 1/(1 + exp(beta04)); !t4 is replaced by beta04 - LC4

odds1 = p1/(1-p1); !LC1

odds2 = p2/(1-p2); !LC2

odds3 = p3/(1-p3); !LC3

odds4 = p4/(1-p4); !LC4

!ref = latent class 2

or12 = odds1/odds2;

!No sysmmetric CI for or12 based on the estimate and SE of

!log or12 = log odds1 - log odds2 = -t1 - (-t2)

or32 = odds3/odds2;

or42 = odds4/odds2;

!ref = latent class 3

or13 = odds1/odds3;

!No sysmmetric CI for or12 based on the estimate and SE of

!log or12 = log odds1 - log odds2 = -t1 - (-t2)

or23 = odds2/odds3;

or43 = odds4/odds3;

!ref = latent class 4

or14 = odds1/odds4;

or24 = odds2/odds4;

or34 = odds3/odds4;

diff12 = -beta01 + beta02;

!diff12 is the same for gender = 1, age and tsdi because

!the slope cancels out

diff32 = -beta03 + beta02;

diff42 = -beta04 + beta02;

OUTPUT:

CINTERVAL;
